# Lichen‐Spinulosus Mimicker: Disseminated and Recurrent Infundibulofolliculitis With Atypical Presentation—A Case Report

**DOI:** 10.1155/crdm/3419730

**Published:** 2026-11-14

**Authors:** Anfal Felimban, Nouf Albarnawi, Raneem Alqahtani, Waseem Alhawsawi, Khalid Alhawsawi

**Affiliations:** ^1^ College of Medicine King Saud Bin Abdulaziz University for Health Sciences Jeddah Saudi Arabia ksau-hs.edu.sa; ^2^ King Abdullah International Medical Research Center Jeddah Saudi Arabia kaimrc.med.sa; ^3^ King Abdulaziz Medical City Jeddah Saudi Arabia mngha.med.sa; ^4^ Department of Dermatology King Abdulaziz Medical City Jeddah Saudi Arabia mngha.med.sa; ^5^ Department of Dermatology King Fahad Armed Forces Hospital Jeddah Saudi Arabia kfafh.org; ^6^ Health Affairs at Ministry of National Guard King Abdullah Specialized Children’s Hospital (KASCH) at King Abdulaziz Medical City Jeddah Saudi Arabia

**Keywords:** case report, chronic folliculitis, disseminate and recurrent infundibulofolliculitis, DRIF, follicular disorders, papular skin eruptions

## Abstract

This case report represents a seven‐year‐old boy with disseminate and recurrent infundibulofolliculitis (DRIF) highlighting the importance of clinicopathological correlation in establishing the diagnosis particularly in pediatric patients. The literature review included multiple studies reporting different treatment options such as vitamin A, topical agents, and phototherapy but with no clear guidelines yet. More case reports and studies are needed to improve understanding and developing standard treatment plans for DRIF.

## 1. Introduction

Disseminate and recurrent infundibulofolliculitis (DRIF) is a rare, chronic, follicular disorder characterized by recurrent papular eruptions originating from the infundibulum of hair follicles. It was first described by Hitch and Lund in 1968, also known as Hitch and Lund disease [1]. It is characterized by multiple pinpoints, skin‐colored, folliculocentric papules resembling “goose bumps” commonly affecting the trunk, neck, buttocks, and arms. The lesions may persist from weeks to years. It is mostly observed among young individuals, with darker skin types (Fitzpatrick IV–VI) and living in hot, humid climates [2–5].

DRIF is considered a dilemma due to its unclear pathogenesis and clinically overlaps with other follicular disorders [3, 4]. While the etiology remains unclear, most researchers describe it as disruption in the keratinization of the follicular infundibulum—the upper part of the hair follicle. This results in unusual retention of keratin, leading to blockage and formation of the characteristic papules. The genetic or familial susceptibility is not supported by the literature [6, 7].

Histopathology is the hallmark for the diagnosis. Edema and lymphocytic infiltration around the infundibulum of the hair follicle are the hallmark for the diagnosis as published in multiple literatures. There is widening and proliferation of cells in the upper part of the follicle [2–4, 8].

There is no standardized treatment of DRIF, but therapies aimed at reducing inflammation. Oral vitamin A, topical keratolytics, psoralen plus ultraviolet A (PUVA) and systemic retinoid have shown benefits [5, 8, 9]. We present this case due to the rarity of the condition and to correlate the clinical presentation with the histopathological features and to highlight the diagnostic features that aid in refining the differential diagnosis.

## 2. Case Presentation

A healthy seven‐year‐old boy presented at the dermatology clinic with a 5‐month history of persistent, slowly progressive skin lesions. The lesions were largely asymptomatic, though the patient had reported transient mild burning pain when the affected skin came into contact with water, with no other identified triggering or relieving factors. Past medical history and review of systems were unremarkable. The parents are not consanguineous and have no history of similar conditions in the family. Skin examination revealed multiple grouped and scaly skin colored follicular papules scattered across the trunk and limbs (Figure 1). Examination of the hair, nails, and mucous membrane was unremarkable. Dermoscopic examination was not performed. The differential diagnosis includes lichen spinulosus, phrynoderma (vitamin A deficiency), pityriasis rubra pilaris (PRP), keratosis pilaris (KP), and keratosis pilaris atrophicans (KPA). A skin biopsy was obtained. Histopathological examination revealed perifollicular edema with the presence of mononuclear cellular infiltrates (Figure 2). Serum vitamin A level was within normal limits 46 mcg/dL, reference range: 20–60 mcg/dL. Based on the above clinicopathological findings, a diagnosis of DRIF was made. The patient was started on oral vitamin A, 5000 IU twice daily for 3 months, topical urea 20% cream twice daily, and topical lactic acid 12% cream twice daily and put under periodic follow up.

**FIGURE 1:**
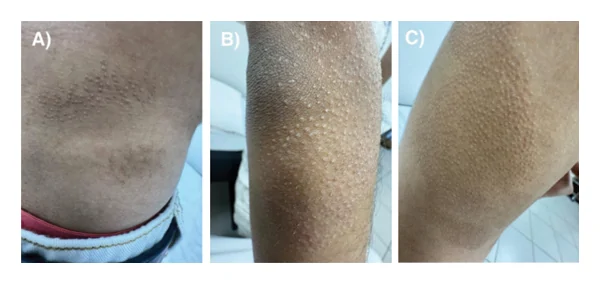
Multiple skin‐colored papules present over the trunk (A). Multiple skin‐colored papules present over the extremities (B and C).

**FIGURE 2:**
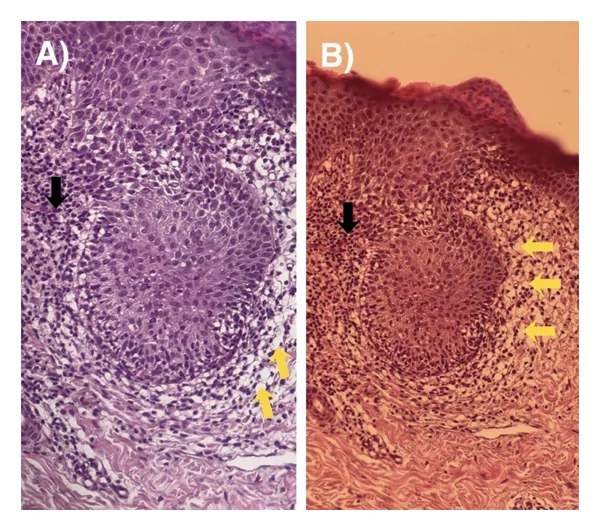
Perifollicular edema (yellow arrows) with the presence of mononuclear cellular infiltrates (black arrow). (A) H&E × 400. (B) H&E × 100.

## 3. Discussion

DRIF is a benign self‐limited disease that is rarely seen in routine dermatology clinics [1]. DRIF can be difficult to diagnose.

The main confirmative diagnosis is histopathological examination [6, 7, 10]. Establishing the diagnosis is often challenging, as DRIF may clinically overlap with several follicular disorders, including lichen spinulosus, phrynoderma, PRP, KP, and KPA. Literature has consistently highlighted this diagnostic difficulty [1–3, 5, 9, 11].

In the differential diagnosis, lichen spinulosus typically presents with grouped follicular papules bearing central spines, while histopathology shows follicular plugging of the infundibulum with perifollicular and perivascular mononuclear infiltrates [12]. Unlike DRIF, there is no infundibular edema. Phrynoderma, linked to vitamin A deficiency, presents as dry, rough papules over extensor surfaces with hyperkeratosis and perifollicular plugging [13]. In this case, normal vitamin A levels helped to exclude phrynoderma, as well as histologically, phrynoderma lacks the characteristic edema of DRIF. PRP may mimic DRIF clinically with follicular papules, but PRP usually includes palmoplantar keratoderma [14]. Histologically, PRP shows alternating ortho‐ and parakeratosis (checkerboard pattern), which was absent here [14]. KP presents with superficial, dry papules commonly on extensor extremities (goosebumps or chicken skin), contrasting with the papules of DRIF involving the trunk and proximal arms [15]. Histologically, KP reveals follicular keratin plugging and hyperkeratosis, which is a hallmark of KP, along with acanthosis and hypergranulosis, perifollicular lymphocytic infiltrates [16]. Finally, KPA shows progressive follicular atrophy and scarring alopecia, often affecting the face and scalp [17]. In our case, no facial involvement and scarring was seen, and histology instead demonstrated no perifollicular scarring.

The dermoscopic and histopathological findings may overlap among different follicular keratotic dermatoses, highlighting the importance of clinicopathological correlation in distinguishing DRIF from its clinical mimickers [18]. Therefore, this case adds to the existing knowledge of DRIF by further expanding the clinical and histopathological spectrum of this rare disorder. A comparison of the clinical, dermoscopic, and histopathological features of DRIF and its differential diagnosis presented in Table 1. While dermoscopy was not performed on our patient, the typical reported dermatoscopic features of DRIF include perifollicular white areas/white halos, sometimes linearly arranged, ill‐defined, or sharply demarcated without prominent follicular plugs [7]. Histopathological examination was therefore central in confirming the diagnosis, as it revealed perifollicular edema with perifollicular inflammatory infiltration, a benchmark feature of DRIF, aligning with previously reported cases [1, 2, 4, 7, 9, 11, 12].

**TABLE 1:** Clinical, dermoscopic, and histopathological features of DRIF and its differential diagnoses.

Differential diagnoses	Clinical features	Dermoscopic features	Histopathological features
Disseminated and recurrent infundibulofolliculitis (DRIF)	Recurrent, asymptomatic, or mildly pruritic, skin‐colored follicular papules over the trunk, neck, and proximal extremities.	Pink, round structures with infiltrative appearance originating around the follicular openings.	Perifollicular edema with a mild inflammatory infiltration of lymphocytes within the follicular infundibulum.
Lichen spinulosus	Grouped, symmetrical follicular papules with a central keratotic spine over the neck, trunk, buttocks, thighs, and extensor extremities.	Perifollicular scaling and white halo, follicular plug, normal interfollicular area [11].	Follicular plugging of the infundibulum with perifollicular and perivascular mononuclear infiltrates.
Phrynoderma	Dry, rough follicular hyperkeratotic papules over extensor surfaces associated with vitamin A deficiency.	Perifollicular scaling with gray–white halo indicative of fibrosis, keratin plug and hyperpigmentation with prominent skin markings of the interfollicular area [11].	Dilated follicular infundibulum filled with keratin debris [11].
Pityriasis rubra pilaris (PRP)	Follicular hyperkeratotic papules, palmoplantar keratoderma.	Follicular plugging and erythema.	Alternating orthokeratosis and parakeratosis in a checkerboard pattern.
Keratosis pilaris (KP)	Superficial, dry papules over extensor extremities (goosebumps or chicken skin).	Perifollicular scaling and erythema with coiled and twisted hair [11].	Follicular keratin plugging, hyperkeratosis, hypergranulosis, perifollicular lymphocytic infiltrates.
Keratosis pilaris atrophicans (KPA)	Progressive follicular atrophy and scarring alopecia, often affecting the face and scalp.	Follicular keratotic plugs, coiled hairs, perifollicular erythema, and areas of atrophy/scarring.	Follicular hyperkeratosis with perifollicular inflammation and progressive follicular destruction.

The patient was subsequently treated with oral vitamin A (5000 IU twice daily) for 3 months along with a topical regimen of 20% urea and 12% lactic acid cream. The rationale of using vitamin A in DRIF comes from the fact that vitamin A regulates epidermal and follicular differentiation and reduces abnormal follicular keratinization. Although the recommended dose of vitamin A in adults is 50,000 IU BID, this is very high for children. We used the National Institutes of Health/Food and Nutrition Board (NIH/FNB) tolerable upper intake levels for preformed vitamin A, which is 5000 IU BID [2, 11, 19]. While literature describes alternative therapeutic approaches—such as the use of UVB phototherapy combined with topical tacrolimus [5, 8] or oral doxycycline [6].

Literature review revealed that despite several isolated case reports, a consistent diagnostic and treatment protocols are not sufficient. Over the past decade, a handful of published cases have contributed to the evolving understanding of this rare condition, offering insights into its clinical presentation, histopathology, and therapeutic outcomes [5, 8, 9].

In 2017, Nair et al. reported a case involving a 17‐year‐old Indian male who presented with monomorphic follicular papules affecting the upper trunk and limbs. Histological examination revealed dilated follicular infundibula with perifollicular lymphocytic infiltration. The patient responded well to narrowband UVB phototherapy in conjunction with topical tacrolimus, suggesting potential benefit from light‐based and immune‐modulating therapies [5].

A case series published in 2019 by Rekha et al. described three Indian patients—two males and one female—aged between 18 and 45 years. These individuals, residing in hot and humid climates, exhibited classic DRIF lesions involving the neck, chest, and upper back. Histopathology consistently showed infundibular plugging with mononuclear cell infiltrate concentrated, attacking the infundibulum of the hair follicle in all cases. The study highlighted diagnostic pitfalls due to overlapping features with KP and follicular eczema, reinforcing the importance of histological confirmation. The author concluded that the perifollicular mononuclear inflammatory cells infiltrate with infundibular cuffing pattern is the only specific and consistent histopathological finding in all cases of DRIF. Other features such as dilated follicle and fibrinoid necrosis are considered associated supportive findings. On the other hand, spongiosis and exocytosis are extremely variable features among the cases and play minor roles in the diagnosis. Cases showed response and improvement with the use of high‐dose vitamin A and a combination of vitamins A and E as well as topical steroids [9].

Shakoei et al. reported a good response of DRIF to antibiotics doxycycline but the need of a very long time to provide temporary relief is doubtful as described by the author due to the nature of this self‐limited condition [6].

Alkul et al. reported a unique case of a middle‐aged man complaining of itchy perifollicular papules around hair follicles on his trunk for 15 years, when he traveled to Afghanistan. The condition usually gets worse as the patient gets sweaty in a hot climate [10].

In addition, a focused review in 2021 discussed therapeutic strategies for DRIF. Drawing on existing literature and clinical observations, they summarized responses to various treatments including topical keratolytics, vitamin A derivatives, calcineurin inhibitors, and phototherapy. The authors stressed the absence of a standardized treatment algorithm and advocated for individualized approaches [2]. Goihman‐Yahr et al. published a case that was successfully treated with PUVA therapy [8].

Most recently, a case report published in 2026 described a 33‐year‐old man presenting with diffuse, pruritic, hyperpigmented, nonerythematous papules involving the neck, trunk, and upper and lower extremities. The differential diagnosis included disseminate and recurrent infundibulofolliculitis, KP, pityrosporum folliculitis, follicular eczema, and lichen simplex chronicus, highlighting the importance of clinicopathological correlation in diagnosing rare follicular dermatoses such as DRIF [20].

## Funding

There are no financial resources to fund this case report.

## Ethics Statement

Consent was taken from the patient’s father in privacy. The information will be kept confidential and the data will be retained in a double locked computer and saved anonymously.

## Consent

The authors obtained written consent from the patient’s father for the photographs and medical information to be published online with the understanding that this information may be publicly available. Patient consent form was provided to the journal.

## Conflicts of Interest

The authors declare no conflicts of interest.

## Data Availability

The data that support the findings of this study are available on request from the corresponding author. The data are not publicly available due to privacy or ethical restrictions.
